# Intestinal Flora Composition Determines Microglia Activation and Improves Epileptic Episode Progress

**DOI:** 10.3389/fcimb.2022.835217

**Published:** 2022-03-09

**Authors:** Xiaomi Ding, Jing Zhou, Li Zhao, Mingyue Chen, Shenglin Wang, Ming Zhang, Xiaodong Zhang, Guohui Jiang

**Affiliations:** Department of Neurology, Institute of Neurological Diseases, Affiliated Hospital of North Sichuan Medical College; North Sichuan Medical College, Nanchong, China

**Keywords:** epilepsy, microglia, gut-brain axis, immune tolerance, 16S rDNA, intestinal flora

## Abstract

In response to environmental stimuli, immune memory mediates the plasticity of myeloid cells. Immune training and immune tolerance are two aspects of plasticity. Microglia that are immunologically trained or immunologically tolerant are endowed with a tendency to differentiate into alternative dominant phenotypes (M1/M2). Male C57BL/6 mice (immune-training group, immune-tolerant group, and control group) were used to establish the kainic acid epilepsy model. The seizure grade, duration, latency, hippocampal potential, and energy density were used to evaluate seizures, and the changes in the polarization of microglia were detected by western blot. 16S rDNA sequencing showed that the abundance of Ruminococcus in the immune-tolerant group was the dominant flora. Our research connections Intestinal microorganisms, brain immune status, and epilepsy behavior together. Pro-inflammatory M1 phenotype and anti-inflammatory M2 phenotype mediate and enhance and suppress subsequent inflammation, respectively. We conclude that intestinal microorganisms influence the occurrence and development of epilepsy by regulating the polarization of microglia.

## Introduction

Epilepsy is a chronic disease caused by excessive excitability of brain neurons and sudden abnormal repeated episodes of epileptic discharges, which lead to temporary disorders related to brain function. The pathogenesis of epilepsy is complex and encompasses many factors, such as environment, heredity, and immunity (Balestrini et al., 2021). The treatment of epilepsy may involve drugs, diet, and surgery (Thijs et al., 2019). However, some patients present with treatment-resistant symptoms that cannot be effectively controlled (Hwang et al., 2019). Thus, exploring the mechanism of epilepsy and its treatment remains a research hotspot.

Microglia are immune memory cells in the brain, and their phenotypic changes are important for disease progression. In the epileptic brain, the microglia may be both epileptogenic and antiepileptic. Inhibiting overactivated microglia *via* inducing phenotype switch from the inflammatory M1 to the protective M2 may be a new therapeutic strategy to alleviate epilepsy (Li et al., 2017; Liu et al., 2018; Deng et al., 2020; Liu et al., 2020). M2 microglia generally play a role in the final stage of an epileptic episode. If the M2 phenotype is activated in the early stage of episode to reduce neuroinflammation, it may be provided beneficial effects to the episode progress. Controlling the M1/M2 polarization ratio of microglia may affect the progression or regression of neuroinflammation in the central nervous system.

Innate immune memory is an important mechanism of plasticity in myeloid cells, which occurs in response to environmental stimulation and changes the subsequent immune inflammatory response. Microglia are the main effector cells. The central nervous system has been shown to respond to environmental changes in immune status induced *via* the peripheral administration of lipopolysaccharide (LPS), which can result in immune training or immune tolerance in the brain immune system (Wendeln et al., 2018). Our study demonstrated that immunotrained or immunotolerant microglia tend to differentiate into the alternative dominant phenotype (M1/M2). IL-10 plays an important role in the regulation of central immune state. IL-10 regulates JAK1/STAT3 signaling pathway and inhibits M1-related inflammatory factors (Essandoh et al., 2016). More importantly, IL-10 mediates the transformation of M2 microglia through GSK3b/PTEN axis (Zhou et al., 2017). On this basis, an epilepsy animal model was established, and glial cells continued to differentiate according to the given phenotype. Our study showed that in the immune-tolerant group, microglia with the M2 phenotype were expressed in large quantities during the early stage of the disease, ultimately improving seizure control.

Recently, the brain–intestinal axis has been suggested to be a bridge to explore and treat central nervous system diseases (Morais and Schreiber, 2021). Previous studies suggested that the intestinal flora is closely related to epilepsy. In the early stage of disease, intestinal flora can be used as a biological marker to diagnose epilepsy (Medel-Matus et al., 2018). In the stage of disease development, intestinal flora can affect the development of epilepsy. Epilepsy is an inflammatory disease (Dahlin and Prast-Nielsen, 2019; De Caro et al., 2019). Intestinal microorganism can regulate intestinal barrier function and play an important role in host immune inflammatory pathway. Tight junctions(TJ) are the principal determinants for intestinal barrier function to maintain mucosal permeability (Xiao et al., 2016). Claudins, the major tight junction proteins, are responsible for the regulation of paracellular space (Günzel and Yu, 2013). Alterations in the claudin levels can affect the intestinal barrier integrity (Findley and Koval, 2009).

In this study, 16S rDNA sequence analysis was used to identify the key flora of immune tolerance to improve epileptic seizures, and to guide the treatment plan.

## Material and Methods

### Animals

C57BL/6 mice were purchased from Jiangsu Jinzhihe Biotechnology Co., Ltd. Specific-pathogen-free (SPF) C57BL/6 adult male mice weighing 22–25 g were housed in groups of five per cage under standard conditions, including a 12-h/12-h light/dark cycle, an environmental temperature of 23°C ± 1°C, humidity of 50–60%, and ad libitum access to food and water. SPF C57BL/6 adult male mice were randomly assigned to the following groups (*n* = 40 per group): (1) control group, (2) immune-training group (1× LPS), and (3) immune-tolerant group (4× LPS).

Our study protocol was approved by the Commission Ethics of Experiments on Animals of North Sichuan Medical College (Nanchong, China) [approval number NSMC(A)2021(20)].

### Peripheral Immune Stimulation

The experimental group was administered daily with an intraperitoneal (i.p.) dose of 500 µg/kg body weight of bacterial lipopolysaccharide (LPS, Sigma-Aldrich, CAT#L2880, US). Animals received either four LPS injections for 4 consecutive days (4× LPS), a single LPS injection followed by three vehicle injections on the following 3 days (1× LPS), or four vehicle injections (PBS). After treatment, they were deeply anesthetized using pentobarbital sodium, blood sample was collected from the right ventricle, and the animals were transcardially perfused with ice-cold PBS through the left ventricle. Next, the brains were removed and fresh-frozen on dry ice. Fresh-frozen hippocampal tissue was homogenized using a Precellys Lysing Kit and machine at 10% or 20% (w/v) in homogenization buffer (50 mM Tris pH 8, 150 mM NaCl, 5 mM EDTA) containing protease inhibitors. Fixed brains were maintained in 4% paraformaldehyde (PFA) for 24 h, followed by two changes of 15% and 30% sucrose solution for dehydration, frozen in optimal cutting temperature compound, and finally coronally sectioned at 12-μm sections using a freezing-sliding microtome (Leica).

### Mouse Seizure Models

On the 15th day after LPS injection, we established an epilepsy animal model to avoid the interference of acute inflammation caused by LPS. The mice were anesthetized with 3% isoflurane in an O2mixture (1 L/min) and placed in a stereotaxic instrument (RWD Life Science Co., Ltd., China). For the kainic acid (KA) model, mice received injections of 0.05 μL KA (Sigma-Aldrich, 1 nmol/50 nL) into the right hippocampus area at a rate of 0.01 μL/min, and we waited for 5 min after the injection to prevent reflux. After KA injection, behavior was evaluated and scored based on the modified Racine scale as follows: grade 0, no response; grade 1, staring and reduced locomotion; grade 2, activation of extensors and rigidity; grade 3, repetitive head and limb movements; grade 4, sustained rearing with clonus; and grade 5, generalized tonic clonic seizures (GTCSs) with loss of posture and death (Racine, 1972).

### *In Vivo* Local Field Potential (LFP) Multi-Tetrode Recordings in the Hippocampus

Mice with stable baseline spontaneous recurrent seizures were used for *in vivo* LFP recordings using an OmniPlex^®^ D neural data acquisition system (Plexon, Dallas, TX, USA) (n=10 per group). To record LFPs, two stainless steel screws into the anterior cranium and a U-shaped frame for holding the head were cemented to the skull, and a microwire array (25 μm in diameter, 16-channel, Yisikepu, China) for LFP recording was implanted into the left hippocampus. LFP activity was continuously recorded and digitized at 4 kHz and filtered (0.1–1,000 Hz) and pre-amplified (×1,000) for 30 min after the baseline was stabilized. For each recording session, we analyzed the periods of sustained epileptic discharges. The epileptiform-like discharge events were analyzed using NeuroExplorer^®^ v5.0 (Plexon, Dallas, TX, USA).

### DNA Sequencing

Total genomic DNA was extracted from the samples using the CTAB/SDS method (n=5 per group). DNA concentration and purity were monitored on a 1% agarose gel. According to the concentration, DNA was diluted to a concentration of 1 ng/μL with sterile water. The following primers were used: 16S V3-V4, 341F-806R; 18S V9, 1380F-1510R; and ITS1, ITS1F-ITS2R. 16S and 18S rRNA genes were amplified using the specific primer with the barcode. All PCR reactions were conducted in a 30 μL reaction mixture with 15 μL of Phusion^®^High-Fidelity PCR Master Mix (New England Biolabs, Ipswich, MA, USA), 0.2 μM each of forward and reverse primers, and approximately 10 ng of the template DNA. Thermal cycling included initial denaturation at 98°C for 1 min, followed by 30 cycles of denaturation at 98°C for 10 s, annealing at 50°C for 30 s, extension at 72°C for 60 s, and final extension at 72°C for 5 min. The same volume of 1× loading buffer (containing SYB green) with PCR products and electrophoresis was loaded onto a 2% agarose gel for detection. Samples with a bright main strip sized 400– 450 bp were selected for further analyses, and the PCR products were mixed in equidensity ratios. Then, the PCR products were purified with the AxyPrepDNA Gel Extraction Kit (Axygen Biosciences, Union City, CA, USA). Sequencing libraries were generated using the NEB Next^®^ Ultra™ DNA Library Prep Kit for Illumina (New England Biolabs, USA) following the manufacturer’s instructions, and index codes were added. The library quality was assessed on a Qubit 2.0 Fluorometer (Thermo Fisher Scientific Inc., Waltham, MA, USA) and Bioanalyzer 2100 system (Agilent Technologies, Inc., Santa Clara, CA, USA). Finally, the library was sequenced on the Miseq/HiSeq2500 platform (Illumina, Inc., San Diego, CA, USA), and 250 bp/300 bp paired-end reads were generated.

### Enzyme-Linked Immunosorbent Assay (ELISA)

To measure blood cytokine levels, serum samples were obtained by coagulating whole blood in EDTA tubes for 10 min at room temperature (i.e., 25 ± 1°C) and centrifugation for 10 min at 2,000 × g (n=6 per group). ELISA kits for mouse IL-10 (ab255729, Abcam Plc, Cambridge, UK) and mouse IL-1 beta (ab197742, Abcam Plc, Cambridge, UK) were used to detect inflammation levels performed according to the manufacturer’s instructions. Each sample was examined at least twice and analyzed using the ELISAcalc software (V0.1, Blue Gene).

### Evaluation of Neuronal Degeneration

Neuronal degeneration was evaluated by Fluoro-Jade B (FJB) staining (n=3 per group). The mice were anesthetized with 3% isoflurane in an O2mixture (1 L/min), immediately followed by 4% PFA, and their brains were fixed in PFA for 24 h. The brains were then successively placed in a 15% and 30% sucrose solution for dehydration and cryosectioned into coronal sections. Frozen sections were dried at 50°C for 30 min and soaked in 80% alcohol containing 1% NaOH for 5 min, followed by immersion in 70% alcohol for 2 min. After washing in distilled water for 2 min, the sections were transferred to a 0.06% potassium permanganate solution for 10 min at room temperature and rinsed in distilled water for 2 min, and 0.004% FJB dye solution (Chemicon International, Temecula, CA, USA) was added for staining in the dark at room temperature for 20–40 min. The slices were washed in distilled water three times for 1 min each, dried with a hairdryer, and immersed in dimethylbenzene for 5 min. Neutral balsam was used to seal the slices. Photos of FJB-positive cells in the hippocampal region (three mice per group and one tissue slice per mouse) were captured using a fluorescence microscope (Olympus, Tokyo, Japan). The excitation wavelength was 488 nm (green), and the emission light was detected using a 520 nm band-pass filter. ImageJ (US National Institute of Health, Bethesda, Maryland, USA) was used to calculate the number of positive cells.

The frozen sections prepared above were used for Iba1 immunofluorescence staining. Transfer slides to a container and cover with antigen retrieval solution according to the table above. Heat slides in a microwave on medium power for 10min. Allow slides to cool in the buffer for 35min. Rinse slides 3 times with 1XTBS for 5min each. Block the sections at room temperature for 1 hour in Blocking buffer. Incubate sections with rabbit anti-Iba1 polyclonal antibody (1:250, 10904-1-AP, Proteintech) in 1XTBS for 2 hours at room temperature or overnight at 4°C. Rinse slides 3 times with 1XTBS for 5min each. Incubate sections with secondary antibody in 1XTBS for 1 hour at room temperature, protected from light. Rinse slides 3 times with 1XTBS for 5min each. Examine slides under a fluorescent microscope (Olympus, Tokyo, Japan). The excitation wavelength was 488 nm (green), and the emission light was detected using a 520 nm band-pass filter.

### Western Blot Analysis

Mice (n = 5 per group) were euthanized 24 h after KA injection. Their brains were excised immediately after they were anesthetized by 3% isoflurane in an O2mixture (1 L/min), and the hippocampal tissue was isolated, homogenized in radioimmunoprecipitation assay buffer (P0013E, Beyotime Biotechnology, Shanghai, China) containing a protease inhibitor, and centrifuged for 15 min at 12,000 rpm and 4°C to collect the supernatant. Protein concentration was measured using a bicinchoninic acid protein assay kit (P0010, Beyotime Biotechnology) according to the manufacturer’s instructions. Equal amounts (30 μg) of total protein were separated on 10% sodium dodecyl sulfate- polyacrylamide gel electrophoresis gels, transferred onto polyvinylidene difluoride membranes (Pall Corp., East Hills, NY, USA) blocked with 5% skim milk for 2 h, and incubated overnight at 4°C with the following primary antibodies: rabbit anti-iNOS polyclonal antibody (1:500, PA1-036, Thermo Fisher), rabbit anti-Arg-1 polyclonal antibody (1:1000, PA5-85267, Thermo Fisher), rabbit anti-claudin-5 polyclonal antibody (1:1000, #29235, SAB, Santa Cruz Biotechnology, Inc., Dallas, TX, USA), CD68 polyclonal Antibody (1:1000, PA5-78996, Thermo Fisher), CD86 polyclonal Antibody (1:1000, PA5-79009, Thermo Fisher),rabbit anti-Iba1 polyclonal antibody(1:1000, PA5-88519, Thermo Fisher), rabbit anti-IL-10 polyclonal antibody (1:500,ab9969,Abcam), GAPDH Rabbit Monoclonal Antibody (1:1000, AF1186, beyotime), and α-Tubulin Rabbit Polyclonal Antibody (1:1000, AF0001, beyotime). The next day, the membrane was washed three times with TBST (Tris-buffered saline with Tween-20) and incubated with secondary antibodies for 1 h at room temperature. Chemiluminescence system (Fusion FX7, Vilber Lourmat, France) was used for protein band visualization and normalized to GAPDH or TUBULIN intensities. The normalized grayscale was obtained by ImageJ software analysis. (US National Institute of Health).

### Statistical Analysis

All statistical analyses were performed using the SPSS statistical software (v. 17.0; IBM Corporation, Armonk, NY, USA). The results are expressed as the mean ± standard deviation. One-way analysis of variance (ANOVA, multiple groups) followed by the Bonferroni test (a correction method for *post hoc* multiple comparisons) was used to compare the differences between the experimental and control groups. The Kruskal–Wallis (K–W) test was performed to compare multiple groups of hierarchical data, and the Mann–Whitney U test was performed to evaluate the difference between the two sets of scored data. Statistical significance was set at *P* < 0.05.

## Results

### Instablish Immune Memory

**Figure 1:**
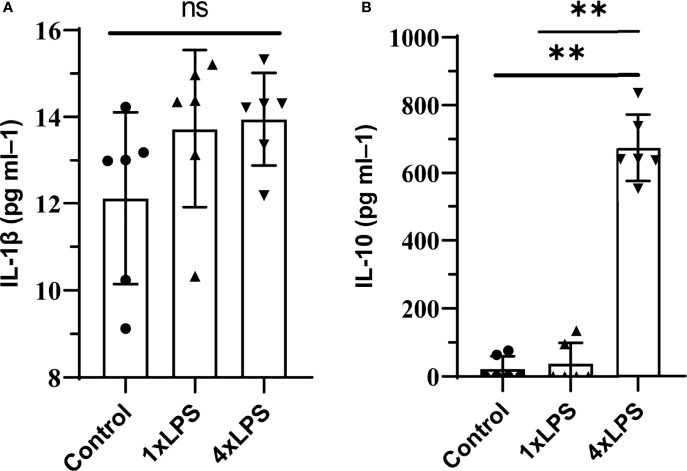
Peripheral cytokine levels in C57BL/6 mice following lipopolysaccharide (LPS) injections. Note that tolerance is induced with repeated injections. IL-1β was detected on day 4, no differences were found. tolerance occurs after immune tolerance (4×LPS). **(A)** There was no significant difference in serum inflammatory factor IL-1β among the three groups. **(B)** The level of IL-10 in the immunotolerant group (4 x LPS) was significantly increased. ns, no significance. **P < 0.01 by one-way ANOVA (and nonparametric or mixed) followed by methods of multiple comparisons.

Firstly, the model of immune training and immune tolerance was established. Then we measured the level of cytokines in peripheral blood on the 4th day after intervention, and found that the serum IL-1 β level of mice in the three groups was low and there was no statistical difference (**Figure 1A**). Compared with the immunological training group and the control group, the level of IL-10 in the immune tolerance group (4 x LPS) increased (P < 0.01, P < 0.01, **Figure 1B**), suggesting the occurrence of immune tolerance.

### Peripheral Immune Stimulation Modulates Epileptic-Seizure Activity

**Figure 2:**
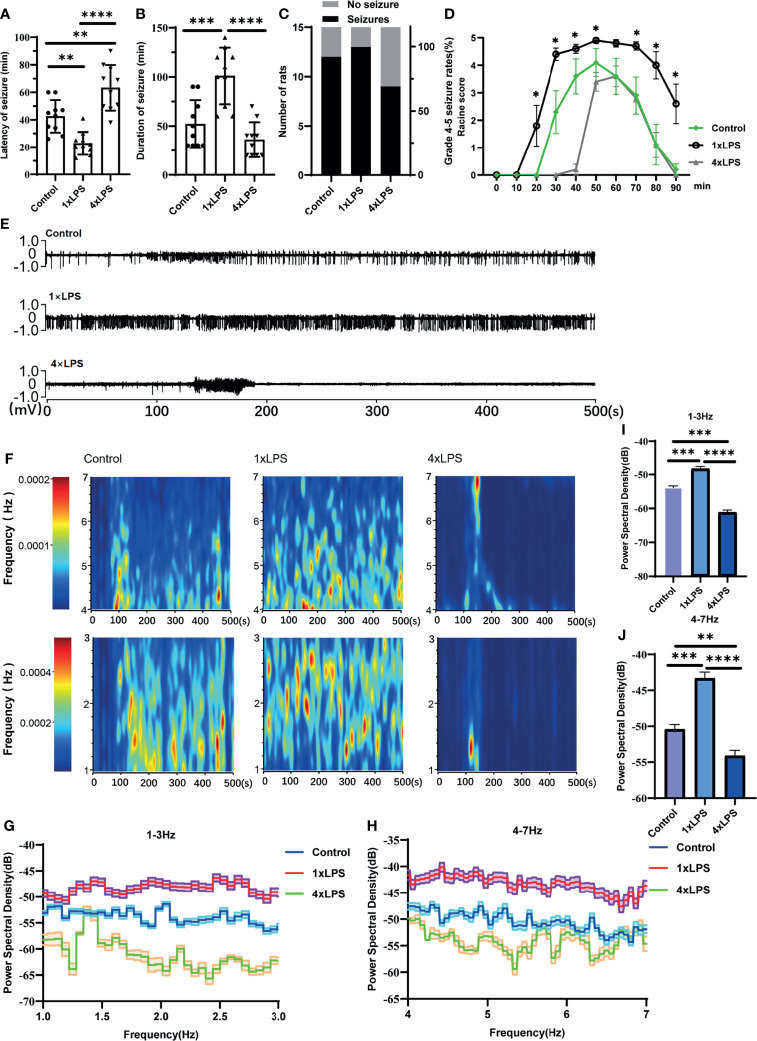
Peripheral immune stimulation modulates epileptic-seizure activity. **(A)** Immune training (1×LPS) and immune tolerance (4×LPS) on the latency of grade 4 or above epileptic seizures. **(B)** The duration of epileptic seizures. **(C)** The proportion of mice with stage 4 or 5 seizures. **(D)** The Racine score at different time points. **(E)** Representative traces of in vivo hippocampal electroencephalogram recordings at 1h after kanic acid injection. **(F)** Corresponding power spectrograms of each group. **(G)** 1-3Hz Energy spectrum trend curve. **(H)** 4-7Hz Energy spectrum trend curve. **(I)** Immune tolerance significantly reduced the average energy density of 1-3Hz oscillatory activity compared to the control group and immune training (1×LPS) group. **(J)** Immune tolerance (4×LPS) significantly reduced the average energy density of 4-7Hz oscillatory activity compared to the control group and immune training (1×LPS) group. *p<0.05; **p < 0.01; ***p < 0.001; ****p < 0.0001 by one-way ANOVA (and nonparametric or mixed) followed by methods of multiple comparisons.

To examine whether changes in peripheral immune stimulation modulate seizures and epileptiform discharges, behavioral tests and LFP measurements were performed. Compared with the control group, the incubation period of KA-induced epilepsy was significantly shortened in the immunotraining group, and the seizure incubation period was significantly prolonged in the immunotolerant group (P < 0.01, P < 0.01, **Figure 2A**). Compared with control group and immune tolerance group, the duration of epileptic seizures above grade 3 in immune training group was significantly longer (P < 0.001, P < 0.0001, **Figure 2B**). Although the duration of attacks above grade 3 in immune tolerance group tended to shorten, there was no difference compared with the control group. To verify the effect of immune tolerance on seizure severity, we observed the proportion of seizure stage 4 and 5 in mice. There was no significant difference in grade 4-5 epilepsy events among the three groups (Chi-square test, p=0. 311, **Figure 2C**). From 20min to 90min after KA intervention, Racine score of immune training group was significantly higher than that of control group and immune tolerance group (**Figure 2D**). We recorded frequent, repetitive seizure-like events (SLEs) in the KA model 24 h after KA injection. Compared with the other two groups, the immune-tolerant group had a significantly lower average energy density (**Figures 2E, F**), which reflects the energy expenditure of brain activity. We evaluated LFP activity at 0–30 Hz, including relatively low frequencies in the delta (1–3 Hz) and theta (4–7 Hz) frequency bands. The delta (1–3 Hz) and theta (4–7 Hz) frequency bands of low-frequency oscillations are also of substantial significance in the diagnosis of epilepsy. The value of the delta (1–3 Hz) band energy density was the lowest in the immune-tolerant group (immune tolerance vs. immune training, P < 0.0001; immune tolerance vs. control group, P < 0.001, **Figures 2G, I**). In the immune-tolerant group, the energy density of the theta (4–7 Hz) frequency band was the lowest (immune tolerance vs. immune training, P < 0.0001; immune tolerance vs. control group, P < 0.01, **Figures 2H, J**).

### 16S rDNA Sequencing Results

**Figure 3:**
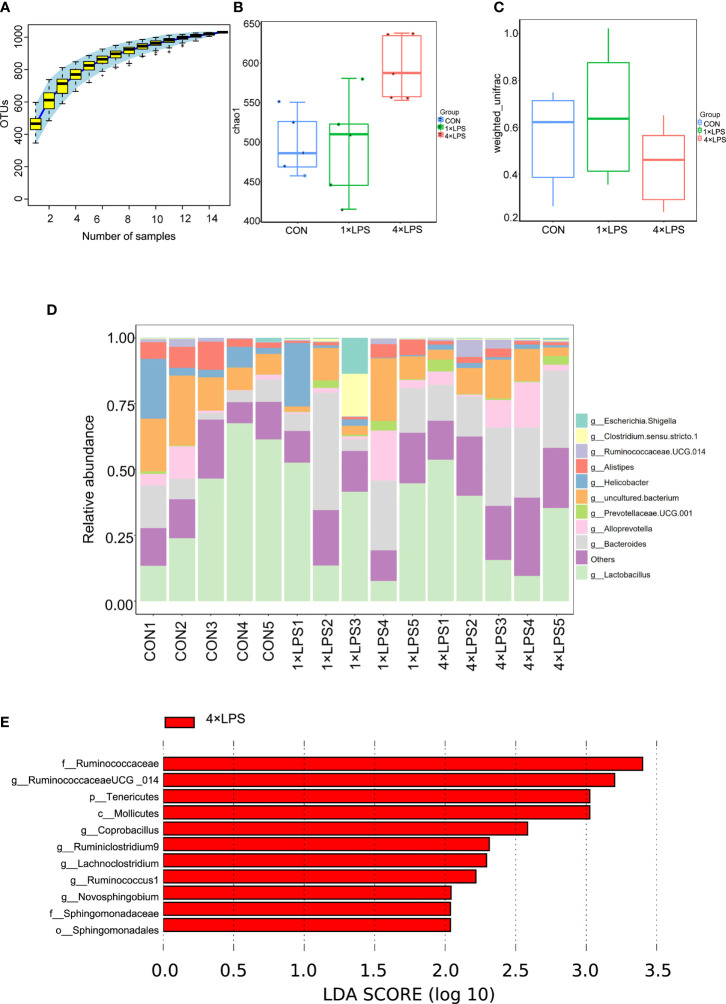
Changes of intestinal flora. **(A)** The upward trend of the box chart of species accumulation curve is stable (n = 5 per group). **(B)** Comparison of microbial genus counts and α-diversity (as assessed by the chao 1) based on the genus profiles in the three groups (Control vs 4×LPS, p<0.05; 1×LPS vs 4×LPS, p <0.01, by t test).**(C)** Comparison of microbial genus counts and β-diversity (as assessed by the unweighted unifrac) based on the genus profiles in the three groups (1×LPS vs 4×LPS, p < 0.05, by t test). **(D)** Composition ratio of three groups of samples at genus level. **(E)** The red area in the histogram of LDA value distribution indicates the microbial groups that play an important role in 4 × LPS. Only the species whose LDA score is greater than the set value (the default setting is 2) are shown in the graph, and the length of the histogram represents the LDA value. The species whose LDA value is greater than 2 by default are Biomarker with statistical differences between groups. Source data are provided as a Source Data file. 1×LPS: immune training; 4×LPS: immune tolerance; LDA: Linear Discriminant Analysis.

The three groups of intestinal microbial communities were characterized using 16S rDNA sequencing. Species accumulation curves showed that sampling was sufficient for data analysis (**Figure 3A**). The intestinal microbial alpha diversity was significantly higher in the immune- tolerant group than in the immune training and control groups (P < 0.01, P < 0.05, **Figure 3B**), indicating that the development of immune tolerance affected the abundance of the flora species. Beta diversity based on unweighted UniFrac distance showed that the difference in species community between the immunotolerant group and the immunetraining group was significant (P < 0.05, **Figure 3C**). The corresponding abundance histogram of intestinal flora at the phylum level showed that the intestinal flora of normal mice was mainly composed of *Firmicutes* and *Bacteroidetes* (**Figures 3D**, **S1A**–**C**), which is consistent with the literature (Chen et al., 2018). Linear discriminant analysis (LDA) value distribution in LDA effect size (LEFSe) suggests that f_Ruminococcaceae, g_RuminococcaceaeUCG_014, p_Tenericutes, c_Mollicutes, g_Coprobacillus, g_Ruminiclostridium9, g_Lachnoclostridium, g_Ruminococcus1, g_Novosphingobium,f_Sphingomonadaceae, and o_Sphingomonadales play an important role in the immune-tolerant group (**Figures 3E**, **S2C**). Among them, *Ruminococcaceae* occupies an important proportion, and its subordinate g_RuminococcaceaeUCG_014 ranked first. The results of COG function and KEGG analysis were meaningless (**Figures S2A, B**).

### Detection of Intestinal TJ and Systemic Inflammatory State

**Figure 4:**
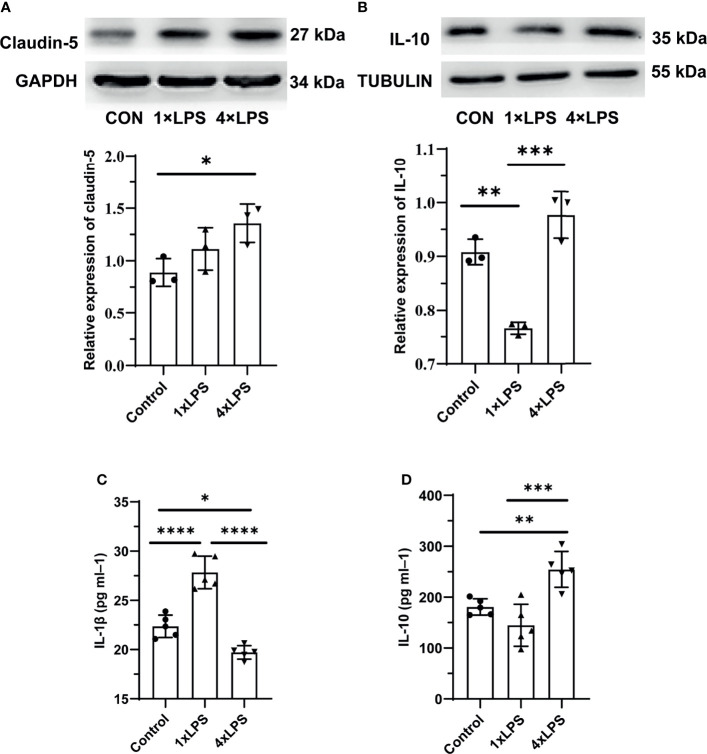
Intestinal barrier and systemic inflammation after establishing epilepsy model. **(A)** Western blot showing protein expression of Claudin-5 protein. Data are expressed as the mean ± SEM (n = 3 per group). **(B)** Western blot showing protein expression of IL-10 protein. Data are expressed as the mean ± SEM (n = 3 per group). **(C)** Serum IL-1β level in 24h after epilepsy induced by kainic acid (n = 5 per group). **(D)** Serum IL-10 level in 24h after epilepsy induced by kainic acid (n = 5 per group). *P < 0.05; **p < 0.01; ***p < 0.001; ****p < 0.0001 by one-way ANOVA (and nonparametric or mixed) followed by methods of multiple comparisons. GAPDH: Glyceraldehyde 3-phosphate dehydrogenase.

In addition to the changes in intestinal flora, we also detected the protein expression of the intestinal tight junction protein (TJ) claudin-5. Compared with the control group, claudin-5 expression increased significantly in the immune-tolerant group (P < 0.05, **Figure 4A**). However, there was no significant difference between the immune training and control groups. In the central nervous system, the levels of IL-10 in hippocampus of control group and immune- tolerant group were significantly higher than those in the immune-training group 24 h after epilepsy modeling (P <0.01, P <0.001, **Figure 4B**). Here, we found that the immune-training group decreased the expression of IL-10. We also examined the concentration of serum inflammatory factors. Compared with the immune tolerance and control groups, the immune-training group showed the highest concentration of the pro-inflammatory factor IL-1β (P < 0.0001, P < 0.0001, **Figure 4C**). Compared with the control group, the immune-tolerant group showed lower concentration of IL-1β (P<0.05, **Figure 4C**). In contrast, the content of anti-inflammatory factor IL-10 in immune tolerance group was the highest, which was significantly different from that in control group and immune training group (P < 0.01, P < 0.001, **Figure 4D**).

### Microglial Activation State

**Figure 5:**
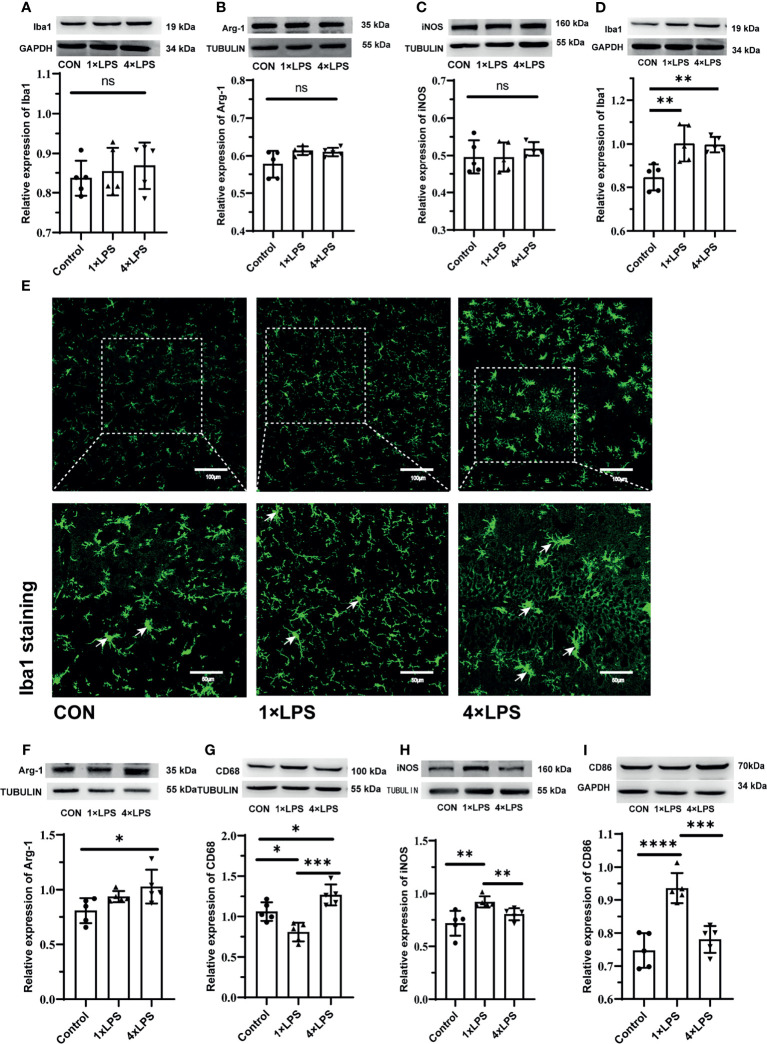
Figures **(A–C)** show the expression and differentiation of microglia before epilepsy modeling. **(A)** Western blot showing protein expression of Iba1 protein. Data are expressed as the mean ± SEM (n = 5 per group). **(B)** Western blot showing protein expression of Arg-1 protein. Data are expressed as the mean ± SEM (n = 5 per group). **(C)** Western blot showing protein expression of iNOS protein. Data are expressed as the mean ± SEM (n = 5 per group). Figures **(D–I)** show the expression and differentiation of microglia 24 hours after epilepsy modeling. **(D)** Western blot showing protein expression of Iba1 protein. Data are expressed as the mean ± SEM (n = 5 per group). **(E)** Iba1 positive cells (green; Alexa Fluor-488 staining) were observed with fluorescence microscope. White arrows indicate Iba1 positive cells. Scale bars: 100 μm for 200×, 50 μm for 400×. **(F)** Western blot showing protein expression of Arg-1 protein. Data are expressed as the mean ± SEM (n = 5 per group). **(G)** Western blot showing protein expression of CD68 protein. Data are expressed as the mean ± SEM (n = 5 per group). **(H)** Western blot showing protein expression of iNOS protein. Data are expressed as the mean ± SEM (n = 5 per group). **(I)** Western blot showing protein expression of CD86 protein. Data are expressed as the mean ± SEM (n = 5 per group). ns, no significance. *P < 0.05; **p < 0.01; ***p < 0.001; ****p < 0.0001 by one-way ANOVA (and nonparametric or mixed) followed by methods of multiple comparisons.

The level of Iba1,Arg-1,iNOS before the establishment of epilepsy model were no statistical difference (**Figures 5A**–**C**). Compared with the control group, the immune-tolerant and immune-training showed increased Iba1 levels 24 hours after epileptic seizure (P < 0.01, P < 0.01, **Figure 5D**). Then microglia were detected using Iba1 immunostaining, which recognizes more activated microglia in the immune-tolerant group and immune-training group(**Figure 5E**). Then, we measured the levels of Arg-1, CD68, iNOS and CD86 in the hippocampus 24 hours after epilepsy modeling. Compared with that in the control group, the concentration of Arg-1 in the immune-tolerant group increased significantly (P < 0.05, **Figure 5F**). Compared with control group and immune-training group, CD68 (another M2 microglia marker) were increased in the immune-tolerant group (P < 0.05, P <0.001, **Figure 5G**). We found that the iNOS levels in the control and immune- tolerant groups were significantly lower than those in the immune- training group (P < 0.01, P < 0.01, **Figure 5H**). CD86, which also represents the activation of M1 microglia, has the same tendency (immune training vs. control group, P < 0.0001, immune tolerance vs. immune training, P < 0.001, **Figure 5I**). As iNOS, CD86, CD68 and Arg-1indicate the presence of the M1 and M2 phenotype, respectively, our results suggest that the immune-training group had dominant M1 phenotype activation, whereas the immune-tolerant group had dominant M2 phenotype activation.

### Evaluation of Neuronal Degeneration and Necrosis

**Figure 6:**
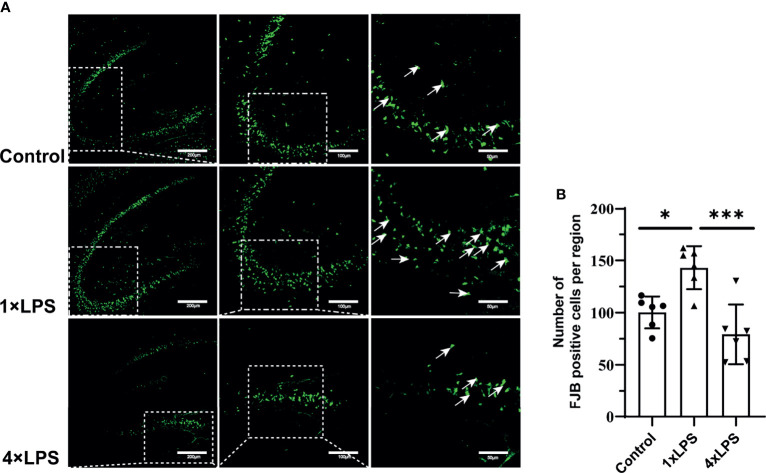
| Immune tolerance alleviates neuronal degeneration after epilepsy. **(A)** FJB positive neurons (green; Alexa Fluor-488 staining) in hippocampus were observed with fluorescence microscope 24 hours after epilepsy modeling. White arrows indicate FJB positive neurons. Scale bars: 200 µm for 100×, 100 µm for 200×, 50 µm for 400×. **(B)** Quantitative statistics of FJB-positive cell number. Data are expressed as the mean ± SEM (n = 3 per group). *P < 0.05; ***p < 0.001 by one-way ANOVA (and nonparametric or mixed) followed by methods of multiple comparisons. FJB: Fluoro-Jade B.

Based on the above results, the degeneration and necrosis of hippocampal neurons were evaluated by FJB staining 24 h after epilepsy modeling. Severely degenerated neurons were found in the hippocampus after epilepsy (**Figure 6A**). The number of FJB-positive cells in the hippocampus of the immune-tolerant group was significantly lower than that of the immune-training group (P < 0.001, **Figure 6B**). Compared with the control group, the immune-training group showed a significantly increased number of FJB-positive cells in the hippocampus (P < 0.05, **Figure 6B**). Therefore, immune tolerance can alleviate the neuronal degeneration caused by epilepsy.

## Discussion

In this study, we investigated whether intestinal microflora can regulate microglial phenotype activation and affect epileptic seizures after inducing immune state change. Immune training and immune tolerance models have been experimented on cerebral ischemia and Alzheimer’s disease models. However, a KA epilepsy mouse model have not been established. Furthermore, this is the first study to investigate the role of the intestinal flora in immune tolerance.

We measured cytokine levels in peripheral serum only after the fourth day of intervention. We found that the release of IL-1β in the serum of 4×LPS-treated mice was very low, but that of IL-10 still increased, indicating the development of immune tolerance (**Figures 1A, B**). For the next two weeks, we fed the mice normally. After 2 weeks, the acute inflammation of mice receded. The level of Iba1, Arg-1, iNOS before the establishment of epilepsy model wereno statistical difference (**Figures 5A**–**C**). This means that there is no difference in the expression of microglia in hippocampus between the three groups after 2 weeks with or without LPS intervention. Then the epilepsy model was established using KA. Behavioral results showed that in the immune-tolerant group, the seizure incubation period was significantly prolonged (**Figure 2A**), the duration of seizure was shortened (**Figure 2B**), and the seizure grade was significantly reduced (**Figure 2D**). Thus, the establishment of immune tolerance may protect against epilepsy. EEG records showed that the frequency of ripple oscillation and energy density were the lowest in the immune-tolerant group, followed by the control group (**Figures 2E, H**). Among them, the δ (1–3 Hz) and θ (4–7 Hz) frequency bands of low frequency oscillation were statistically significant (**Figures 2I, J**). Brain waves with frequencies below 8 Hz are collectively called slow waves. Slow waves have various manifestations in epileptic seizures, including spike-slow wave, multi-spike slow wave, sharp slow wave and other wave types, which are collectively called epileptiform discharges. The spike-slow wave complex rhythm is more common in minor absence seizures (Sitnikova et al., 2021), and a spike-slow wave is the most common in Lennox-Gastaut syndrome (Thiele et al., 2018). Although focal and regional background slowdown (theta and delta) is a nonspecific finding in patients with epilepsy (Jan et al., 2010), rhythmic theta activity is a common attack pattern in temporal lobe epilepsy (TLE) (Rosenow et al., 2015). Thus, our results showed that peripheral LPS injection can change the seizure configuration depending on the treatment period.

However, LPS intervention cannot be currently performed in advance to prevent or treat epilepsy in practical work. Therefore, we attempted to obtain information from the intestinal flora. We collected feces 24 h after epilepsy and outsourced them for 16S rDNA sequencing. As immune status changes, the composition of the intestinal flora also changed rapidly and significantly. 16S rDNA sequencing of intestinal microorganisms showed that f_Ruminococcaceae abundance increased most significantly in the immune-tolerant group (**Figure 3E**). This flora is closely related to nervous system diseases, such as Alzheimer’s disease, depression, and stroke (Chang et al., 2021; Chen et al., 2021; Cuervo-Zanatta et al., 2021). Ruminococcus helps inhibit the translocation of bacterial products (e.g., LPS) into the blood, which may activate the Toll-like receptor 4 (TLR4), stimulate inflammatory responses, and secrete proinflammatory cytokines (Shi et al., 2006; Cani et al., 2007). The microbial flora can produce a large number of metabolites, which can affect the brain (Mayer et al., 2014). Ruminococcus degrades a variety of polysaccharides and fibers to produce short-chain fatty acids (SCFAs) (Hooda et al., 2012; Donaldson et al., 2016). SCFAs, such as butyrate, propionate, and acetate, are a class of bacterial metabolites that have pleiotropic effects on host immunity and energy states. Some studies have shown that the differential flora g_RuminococcaceaeUCG_014 detected is positively correlated with butyric acid concentration in the cecum (Chen et al., 2020). Butyric acid is the main energy source for colonic epithelial cells, which primarily strengthen the physical barrier. Previous studies have suggested that cAMP levels increase in butyrate-containing cell cultures or in the intestinal epithelial cells of butyrate-fed mouse (Ghanim et al., 2009). Therefore, intestinal epithelial cells can use butyrate as a key energy substrate to produce ATP (Chang et al., 2017). At the same time, patients with colitis benefit from butyrate enema, indicating its local anti-inflammatory effects (Guo et al., 2015; Chu et al., 2019). Butyric acid also inhibits histone deacetylase (HDAC) activity in the colon and immune cells (Devaraj et al., 2013; Chen et al., 2018) and can induce an increase in histone H3 acetylation in the promoter and enhancer regions of FOXP3, leading to an increase in FOXP3 expression (Etxeberria et al., 2013). This process affects the differentiation of regulatory T cells (Tregs) and controls intestinal inflammation (Etxeberria et al., 2013; Crovesy et al., 2017; Wang et al., 2020). In summary, Ruminococcus can maintain the intestinal mucosal barrier and protect mucosal tissues from proinflammatory molecules and microorganisms.

The butyrate induces epithelial growth and cellular proliferation in normal intestinal tissue (Parada Venegas et al., 2019). G-protein coupled receptors, including GPR41, GPR43, and GPR109a, are expressed in colon cells. GPR109a interacts with butyrate: the butyric acid-driven signal interactions involved in GPR109a may promote the differentiation of Tregs and IL-10-producing T cells (Singh et al., 2014; Hanus et al., 2021). SCFAs reduce the production of proinflammatory cytokines by inhibiting HDAC and enhancing the expression of tight junction (TJ) proteins (Ma et al., 2012; Lin et al., 2015). Consequently, we confirmed that the levels of intestinal claudin-5 were higher in the immune-tolerant group (**Figure 4A**). This means that the intestinal tract of the immune tolerance group is more powerful to defend against the invasion of endogenous microorganisms and their toxins. The concentration of the anti-inflammatory factor IL-10 was the highest in the immune-tolerant group in the center and periphery compared with other groups (**Figures 4B, D**). In the central nervous system, IL-10 binds to its receptor and activates downstream JAK1, leading to STAT3 translocation into the nucleus. Translocation of STAT3 inhibits most M1-related proinflammatory cytokines (Essandoh et al., 2016). L-10 mediated the polarization of M2 microglia by increasing the expression of Glycogen Synthase Kinase 3 beta (GSK3β) (Zhou et al., 2017).

The expression of Iba1 in the experimental group increased, indicating that more microglia were activated. Indeed, we found that iNOS and CD86 levels in the control and immune-tolerant groups were significantly lower than those in the immune-training group (**Figures 5H, I**). Reduction of M1 phenotype activation alleviates epileptic seizures. The Arg-1 and CD68 concentration, representing the M2 phenotype, increased significantly in the immune-tolerant group (**Figures 5F, G**). In addition, we compared the expression of Arg-1 and iNOS longitudinally. Before and after the establishment of KA model, the expression of Arg-1 in immune tolerance group was the most, while iNOS increased more significantly in immune training group. During immune training, proinflammatory cytokines released by the M1 phenotype participate in proinflammatory and pro- killing processes. In immune tolerance, the M2 phenotype is dominant and performs protection and repair functions. The microglial phenotype is adjustable and responsive to environmental changes (Subramaniam and Federoff, 2017). Based on the above results, we then evaluated the degeneration and necrosis of hippocampal neurons after epilepsy modeling and found that the number of FJB-positive cells in the hippocampus of the immune-tolerant group was significantly lower than that of the immune-training group (**Figure 6B**). The number of FJB-positive cells in the hippocampus of the immune-training group increased significantly compared with that in the control group. Therefore, immune tolerance can alleviate the neuronal degeneration and necrosis caused by epilepsy. Increased levels of Ruminococcus detected in the immune-tolerant group have a profound effect on systemic metabolic status in terms of inflammation.

A recent study found that the expression of CYP3A and P-gp in the intestinal tract of diabetic mice was positively correlated with Lachnoclostridium and unclassified _f_Ruminococcaceae (Hu et al., 2021). CYP3A1 is a mouse gene that has 73% amino acid homology with the human CYP3A4. P-gp is encoded by the ABCB1 gene in humans and the ABCB1A and ABCB1B genes in rodents. Both proteins are related to drug metabolism. Most studies on drug metabolism focus on the liver. However, there are a few studies investigating the relationship between CYP3A and P-gp expression in the intestinal tract and drug metabolism. Antiepileptic drugs have different activating or inhibitory effects on metabolic enzymes. For example, carbamazepine, oxcarbazepine, and phenytoin can induce CYP3A4 activity (Ghosh et al., 2018; Chen et al., 2021; Roberti et al., 2021), whereas levetiracetam, phenobarbital, and phenytoin induce P-gp expression (Jing et al., 2010; Moerman et al., 2011; Alvariza et al., 2014). Follow- up studies can determine whether the metabolism of antiepileptic drugs is related to the expression of CYP3A1 and P-gp in the intestinal tract and whether changes in the intestinal flora of patients with epilepsy have an impact on the expression of CYP3A and P-gp in the intestinal wall and in an epilepsy model. Furthermore, whether the clinically significant expression of CYP3A1 and P-gp in the intestinal tract will lead to a decrease in the therapeutic effect or alleviate the side effects of drugs should be explored.

This study had several limitations. We did not perform a metabonomic analysis of the intestinal contents and did not assess the contents of short-chain fatty acids. The lack of behavioral data of over-excitement is the limitation of our experiment. Moreover, the proposed mechanism underlying the action of key flora was not verified. Nevertheless, this study provides novel insights on intestinal flora regulation through microglial cell phenotype transition and contributes to the development of new clinical interventions in epilepsy. Overall, the intestinal flora can significantly change the immune state, thereby affecting neuronal excitability and improving the progress of epileptic episodes. This study provides valuable insights and experimental findings for the development of new clinical interventions in epilepsy.

## Data Availability Statement

The original contributions presented in the study are publicly available. This data can be found here: https://www.ncbi.nlm.nih.gov/bioproject/, PRJNA790496.

## Ethics Statement 

Our study protocol was approved by the Commission Ethics of Experiments on Animals of North Sichuan Medical College (Nanjing, China) [approval number NSMC(A)2021(20)].

## Author Contributions

All authors agree to be accountable for all aspects of the work in ensuring that questions related to the accuracy or integrity of any part of the work are appropriately investigated and resolved. Conceptualization: XD and GJ. Investigation, Project administration and Writing - original draft: XD and LZ. Formal analysis, Validation and Visualization: MC and SW. Methodology: LZ and MZ. Funding acquisition, Resources, Supervision: JZ and GJ. Writing - review & editing: XZ, JZ, and GJ. All authors contributed to the article and approved the submitted version.

## Funding

This work was supported by the National Natural Science Foundation of China (No. 81971220) and the Nanchong City Cooperative Scientific Research Project in 2019 (North Sichuan Medical College) (No. 19SXHZ0097).

## Conflict of Interest

The authors declare that the research was conducted in the absence of any commercial or financial relationships that could be construed as a potential conflict of interest.

## Publisher’s Note

All claims expressed in this article are solely those of the authors and do not necessarily represent those of their affiliated organizations, or those of the publisher, the editors and the reviewers. Any product that may be evaluated in this article, or claim that may be made by its manufacturer, is not guaranteed or endorsed by the publisher.
