# Supplementary figures and images for: Intestinal Flora Composition Determines Microglia Activation and Improves Epileptic Episode Progress
Source: Front Cell Infect Microbiol. 2022 Mar 9;12:835217. doi: 10.3389/fcimb.2022.835217 (PMC8959590; doi:10.3389/fcimb.2022.835217)

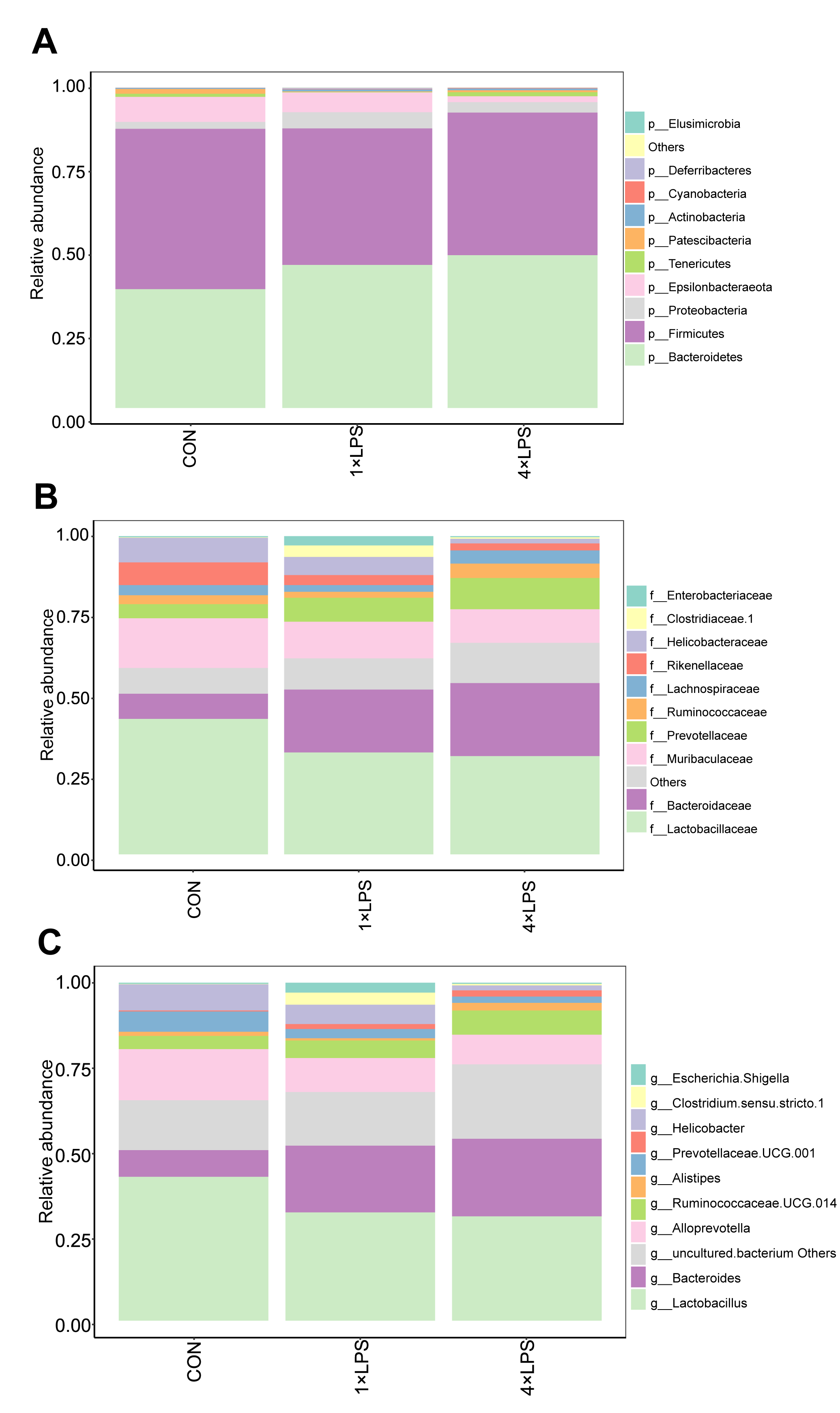

Supplement: Supplementary file 1 [file Image_1.tif]

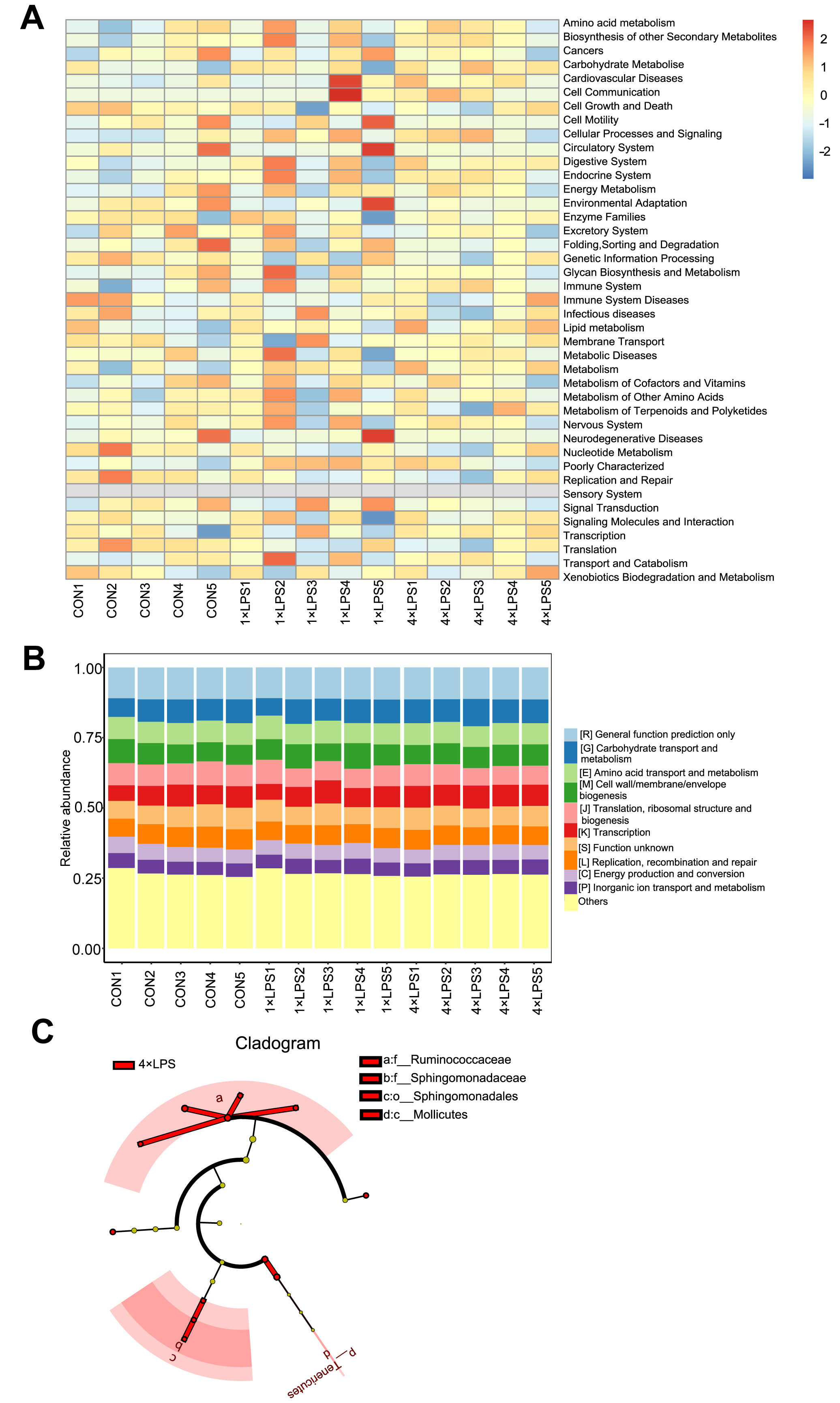

Supplement: Supplementary file 2 [file Image_2.tif]

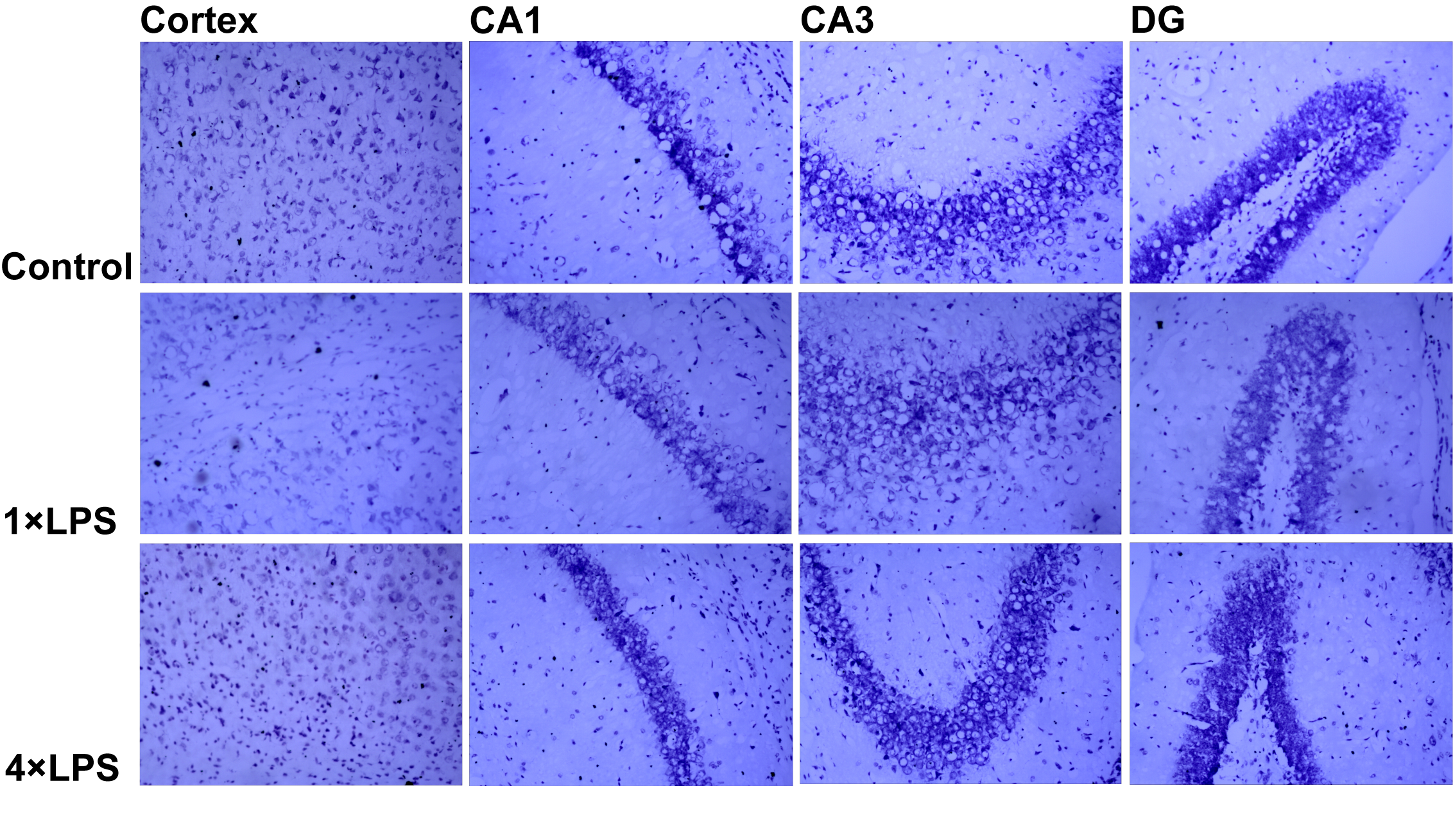

Supplement: Supplementary file 3 [file Image_3.tif]
